# Editorial: Pharmacodynamic and pharmacokinetic aspects of redox signaling in inflammation-associated damage and diseases

**DOI:** 10.3389/fphys.2026.1849826

**Published:** 2026-04-28

**Authors:** Mamunur Rashid, Siva Rama Raju Kanumuri, Mohd Yaseen Malik

**Affiliations:** 1 Department of Pharmacy Practice, University of Nebraska Medical Center, Omaha, NE, United States; 2 Department of Pharmaceutics, University of Florida, Gainesville, FL, United States; 3 Department of Pharmacology, University of Oxford, Oxford, United Kingdom

**Keywords:** inflammatory mediators, oxidative stress, pharmacodynamics, pharmacokinetics, reactive oxygen species (ROS)

## Introduction

Inflammation is a complex cellular process that plays a significant role in tissue repair and host defense. However, prolonged or compromised inflammatory responses led to the development and progression of many chronic diseases, such as respiratory-related disorders, metabolic syndrome, and systemic inflammatory syndrome. Redox signaling molecules, reactive oxygen species (ROS), and reactive nitrogen species (RNS) regulate cellular pathways. Under physiological conditions, these molecules play a vital role as signaling mediators that control cell functions, including proliferation, immune response, and metabolic homeostasis. Normally, cells maintain homeostasis by balancing oxidant and antioxidant systems. Disruption of balance leads to excessive production of reactive species, causing oxidative stress. This may cause cellular dysfunction, tissue damage, and the progression of inflammation-associated diseases.

Understanding the pharmacological modulation of redox signaling has therefore become a key area of biomedical research, focusing on how redox signaling contributes to disease pathology and how therapeutic strategies can restore balance. In this context, both pharmacokinetic and pharmacodynamic factors play an important role in determining efficacy. Pharmacodynamics studies provide insight into how therapeutic agents influence oxidative signaling pathways and inflammatory responses, while pharmacokinetics provides the drug’s absorption, distribution, metabolism, and elimination in the body. Together, PK-PD parameters provide a framework for improving therapeutic strategies aimed at restoring redox homeostasis.

This Research Topic, “*Pharmacodynamic and pharmacokinetic aspects of redox signaling in inflammation-associated damage and diseases*,” compiles a collection of four review articles and couple of original research studies that explore the oxidative stress mechanisms, therapeutic approaches, inflammatory related disease models, and advances in drug delivery methods. Collectively, these studies highlight the importance of redox biology in the pathogenesis of inflammatory-related diseases and underscore emerging pharmacological approaches that target oxidative pathways.

Cazzola et al. contributed a review article on this Research Topic, which focuses on the role of redox biology in chronic airways disease. Chronic respiratory disorders such asthma and chronic obstructive pulmonary disease (COPD), bronchiectasis are characterized by persistent inflammation and oxidative imbalance. The study highlights how dysregulation of ROS/RNS production impairs the antioxidant defense mechanism, leading to airway inflammation, epithelial dysfunction, and immunological dysregulation. Furthermore, the authors emphasized the potential of precision antioxidant treatment methods as adjuncts to conventional therapy by targeting redox-dependent pathways to reduce inflammation, improve respiratory function, and help in managing chronic airways disorders.

Environmental exposure, such as hypobaric hypoxia, promotes oxidative imbalance and deteriorates the endogenous antioxidant defense mechanism. Pena et al. published a comprehensive review and meta-analysis on the role of antioxidant treatment in patients suffering from acute mountain sickness caused by high altitude exposure. High altitude environments are related to hypoxia-induced oxidative stress, which contributes to the pathogenesis of acute mountain sickness. The review provides clinical evidence to support the benefits of antioxidant supplementation in lowering oxidative stress and improving symptoms in affected individuals. The review article highlighted the crucial role of redox modulation in extreme environmental conditions.

Natural compounds with antioxidant properties attract considerable interest in pharmacological research for their ability to modulate oxidative stress. In this context, the review article by Gao, Linran et al.  provides a comprehensive overview of the molecular mechanisms underlying the pharmacological effects of chlorogenic acid. The compound, widely found in many plant species, exhibits significant antioxidants, anti-inflammatory, and metabolic regulatory properties. The review further discusses recent advancements in drug delivery systems to enhance the bioavailability and therapeutic efficacy of chlorogenic acid. Such approaches highlight the importance of pharmacokinetics optimization in the development of natural product-based therapeutics.

Another article focuses on inflammation, a crucial factor of cystic fibrosis in airway epithelial cells. The study by Kouadri et al. evaluated whether the inflammatory response depends on CFTR’S chloride channel transporter or its structural integrity. The study explores the impact of modulators and wild-type CFTR overexpression on CFTR expression, trafficking, chloride function, and inflammation in CF bronchial epithelial cells. The study revealed that overexpression of wild-type CFTR completely restores chloride secretion and normalizes inflammation to non-CF cells, emphasizes the necessity for therapeutic strategies that repair both channel function and CFTR misfolding.

Further, Ruan et al. contributed an original research article exploring the role of inflammation in lung injury and highlighted the protective role of carnosine, a naturally occurring dipeptide with antioxidant benefits. The study demonstrates that carnosine acts as a protective metabolic mediator of inflammatory lung injury by modulating innate immune cell recruitment and macrophage polarization. These results suggest that carnosine may serve as a promising metabolic mediator capable of modulating immune response in inflammatory lung diseases.

Beyond respiratory diseases, oxidative stress and inflammation are linked with a metabolic syndrome (MetS), which includes obesity, dyslipidemia, hypertension, and insulin resistance. A review published by Hamooya et al. in this Research Topic provides a comprehensive overview of its epidemiology, mechanisms, and current dietary and lifestyle-based management. The author emphasized that oxidative stress and inflammation cause MetS, which is linked to increase the risk of cardiovascular disease, stroke, and type 2 diabetes. The study highlights the importance of lifestyle modifications, early screening, and dietary changes is an effective approach for preventing and managing metabolic syndrome.

Taken together, the articles published in this Research Topic highlight the role of redox signaling in inflammation-related disease. The research illustrates that the reactive oxygen species are not merely a byproduct of cellular metabolism but also a key regulator of several biological processes and underlines that targeting redox balance may be a promising therapeutic strategy.

## Challenges and future directions

Future research in this field should focus on further elucidating the molecular mechanisms between redox signaling and its interaction with inflammatory pathways. In addition, the integration of pharmacokinetics and pharmacodynamics modeling helps to bridge the gap between experimental findings and clinical application and will be essential for optimizing the therapeutic efficacy of redox- targeting drugs. Advances in drug delivery technology, system biology approaches, and translational pharmacology may also contribute to the development of more effective treatments for inflammation-related diseases.

## Conclusion

In conclusion, this Research Topic provides a comprehensive overview of current progress in understanding the pharmacodynamics and pharmacokinetic aspects of redox signaling in inflammation-associated disease. The contributions presented here not only advance our understanding of oxidative stress mechanisms but also highlight emerging therapeutic potential in current pharmacological research, which may help guide future efforts to develop innovative treatments for inflammation-driven disorders.

